# Woodhouse-Sakati Syndrome Due to the Rare DCAF17 c.321+1G>A Mutation: The Second Case Report Worldwide

**DOI:** 10.7759/cureus.108419

**Published:** 2026-05-07

**Authors:** Zhahid H Baigh, Jibran A Sheikh, Baleekhudin Mohd O Dawar

**Affiliations:** 1 Internal Medicine, Government Medical College Srinagar, Srinagar, IND; 2 Internal Medicine, Government Medical College Jammu, Jammu, IND

**Keywords:** dcaf17, diabetes mellitus, hypogonadism, intellectual impairment, woodhouse-sakati syndrome

## Abstract

Woodhouse-Sakati syndrome (WSS) is a rare autosomal recessive neuroendocrine disorder caused by pathogenic variants in the DDB1- and CUL4-associated factor 17 (*DCAF17*) gene. The c.321+1G>A splice-site mutation is an extremely rare variant with limited reports in the literature. Identification of such ultra-rare variants contributes to strengthening genotype-phenotype correlations. We describe a 33-year-old Kashmiri woman with classical features of WSS, including hypergonadotropic hypogonadism, diffuse alopecia, diabetes mellitus, characteristic facies, and intellectual disability. Whole-exome sequencing revealed a homozygous *DCAF17* c.321+1G>A variant. This case represents a rare occurrence of this mutation and provides additional evidence supporting its pathogenic role in WSS.

## Introduction

Woodhouse-Sakati syndrome (WSS) is a rare autosomal recessive multisystem disorder caused by pathogenic variants in the DDB1- and CUL4-associated factor 17 (*DCAF17*) gene [1]. It was first described in 1983 by Woodhouse and Sakati in two Saudi families with a combination of hypogonadism, partial alopecia, diabetes mellitus, intellectual disability, and deafness [2].

It is characterized by alopecia, hypogonadism, diabetes mellitus, intellectual disability, extrapyramidal signs (dystonia, dysarthria, choreoathetosis, and spastic quadriplegia), and variable additional features including sensorineural deafness, hypothyroidism, low insulin-like growth factor (IGF-1) levels, electrocardiographic abnormalities and typical facies (flat occiput, triangular face, high forehead, frontal bossing, mild hypertelorism, short and sparse eyebrows, down-slanting palpebral fissures, a prominent nasal root, dental malocclusion, and a high-arched palate). There is no clear age of onset for the disorder, but the different clinical manifestations can present at different times; for example, hypogonadism is often detected around the time of puberty (12-14 years of age), diabetes mellitus and hypothyroidism during adolescence up to 25 years of age, and neurological manifestations between 9 and 17 years of age [3]. Fewer than 200 genetically confirmed cases have been reported worldwide as of 2023 [4].

The DDB1- and CUL4-associated factor 17​​​​​​​ (*DCAF17*) gene encodes a nucleolar protein that is thought to function as a substrate receptor within the DDB1-CUL4 E3 ubiquitin ligase complex, a key component of the ubiquitin-proteasome system. Proteins in this family typically facilitate the recognition and targeting of specific substrates for ubiquitination and subsequent proteasomal degradation, thereby contributing to cellular protein homeostasis [5]. Although the precise biological role of *DCAF17* remains incompletely defined, its localization to the nucleolus suggests a role in nucleolar integrity, ribosomal biogenesis, and RNA processing. Loss-of-function variants in *DCAF17* are believed to disrupt these processes, leading to impaired cellular function in metabolically active and hormonally regulated tissues, particularly endocrine organs and the central nervous system. This likely underlies the multisystem phenotype observed in Woodhouse-Sakati syndrome, including hypogonadism, alopecia, diabetes mellitus, and neurodevelopmental abnormalities.

Here, we report a case with classical features and a specific splice variant *DCAF17* mutation, only once previously described in a family from Pakistan [6].

## Case presentation

A 33-year-old woman, born to first-cousin parents, presented for evaluation of primary amenorrhea, progressive alopecia, and poorly controlled diabetes mellitus. She had lifelong mild intellectual disability, evidenced by difficulty completing formal education (she did not complete high school), although she remained independent in activities of daily living with preserved speech and comprehension. Diabetes mellitus had been diagnosed before the age of 27 years. Alopecia and characteristic facial features had progressed throughout childhood and adolescence. The patient had one older sibling, a brother, who was clinically unaffected. No other family members exhibited a similar clinical phenotype.

Physical examination was notable for a height of 170 cm, weight of 58 kg, and BMI of 20 kg/m². She had a triangular face with a prominent forehead, signs of premature aging, a prominent nasal root, diffuse non-scarring scalp alopecia, and sparse eyebrows and body hair. There was also a very significant deep transverse crease over the forehead. Breast development and axillary hair were rudimentary at Tanner stage 1. There were no skeletal or dental deformities. Laboratory findings are given in Table 1.

**Table 1:** Laboratory findings of the patient at different times ALKP: alkaline phosphatase, ALT: alanine transaminase, AST: aspartate transaminase, FSH: follicle-stimulating hormone, HbA1c: glycated hemoglobin, LH: luteinizing hormone, NT: not tested, TSH: thyroid-stimulating hormone, T3: tri-iodothyronine, T4: thyroxine

	At age 7 years	At present (age 33 years)	Reference range
LH	11.6 mIU/mL	8.32 mIU/mL	<9 mIU/mL (age 7), 1-12 mIU/mL (adult follicular/luteal phase)
FSH	51.2 mIU/mL	42 mIU/mL	<9 mIU/mL (age 7), 2-9 mIU/mL (adult follicular/luteal phase)
Testosterone	20 ng/dL	NT	18-54 ng/dL
Prolactin	3.33 ng/mL	1.89 ng/mL	<20 ng/mL
TSH	15.3 µIU/mL	NT	0.5-4 µIU/mL
Total T4	5.44 µg/dL	NT	5-12 µg/dL
Total T3	121 ng/dL	NT	80-180 ng/dL
HbA1c	NT	13.9%	<5.7%
AST	NT	45 U/L	10-40 U/L
ALT	NT	48.9 U/L	10-40 U/L
Bilirubin, total	NT	0.46 mg/dL	0.3-1 mg/dL
ALKP	NT	100.9 U/L	3-120 U/L
Albumin	NT	4.2 g/dL	3.5-5.5 g/dL

These values demonstrated premature hypergonadotropic hypogonadism and subclinical hypothyroidism.

**Figure 1:**
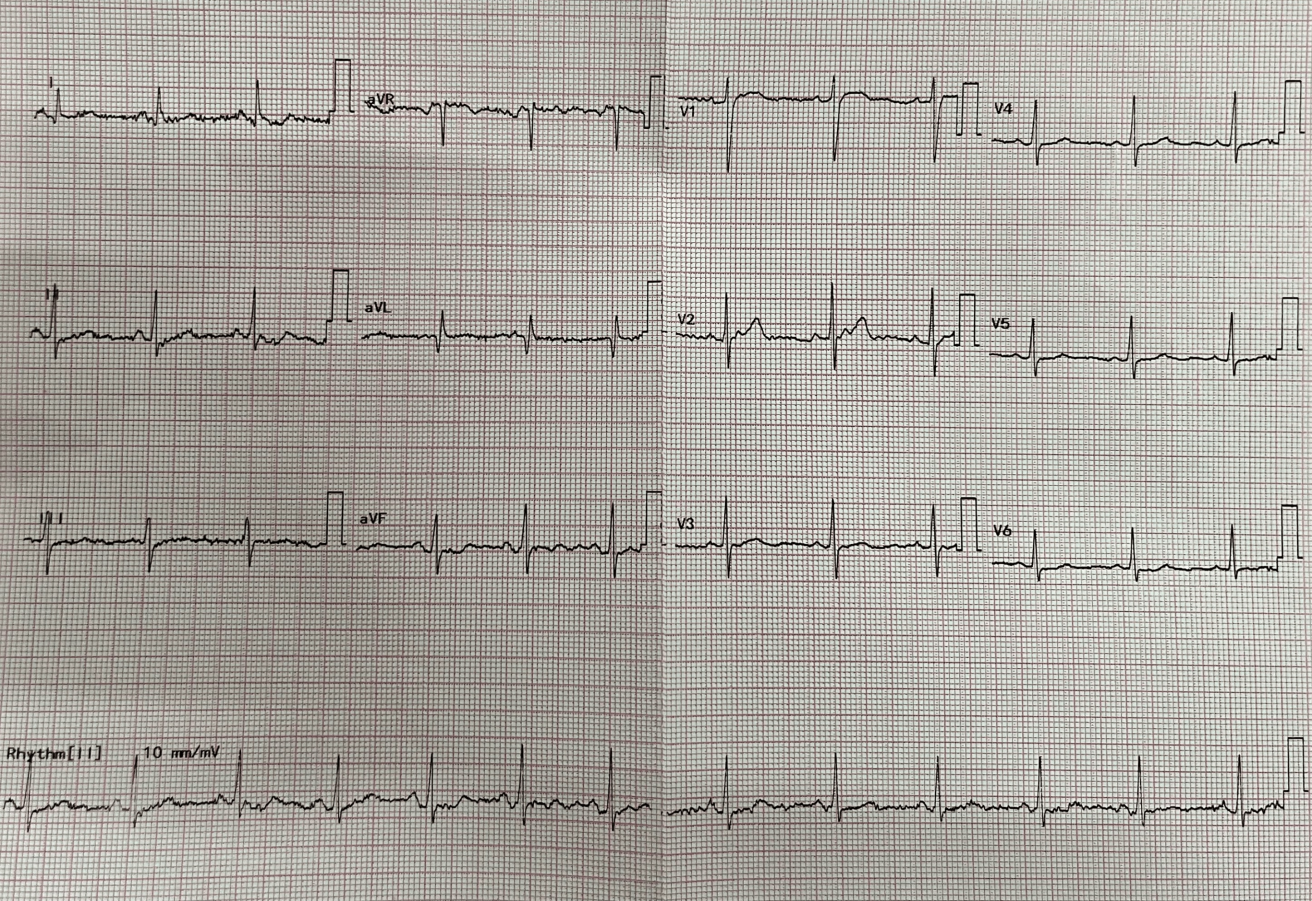
Electrocardiogram of the patient showing normal sinus rhythm

Echocardiogram was unremarkable, and an abdominal ultrasound was notable for the absence of a distinct uterus and adnexal identification. Liver echotexture was described as coarse with grade 2 hepatic steatosis and prominent left hepatic and caudate lobes. Dual-energy X-ray absorptiometry (DEXA) scan of the lumbar spine was suggestive of osteopenia (T scores of -1.7), likely related to the hypoestrogenic state. Electrocardiogram (ECG) showed normal sinus rhythm without any ST and T-wave abnormalities (Figure 1).

Karyotyping revealed a normal 46,XX complement. A presumptive clinical diagnosis of Woodhouse-Sakati syndrome (WSS) was made based on the clinical phenotype of hypogonadism, alopecia, diabetes mellitus, and intellectual disability. Whole-exome sequencing was subsequently performed.

The sequencing revealed a homozygous mutation of c.321+1G>A in the *DCAF17* gene. The same mutation was described by Habib et al. in a family affected with WSS in 2011 [6]. Based on the clinical phenotype and the genetic variant, the diagnosis of WSS was confirmed. Genetic testing of the patient’s parents and brother could not be performed due to financial constraints. Table 2 lists the classic features of the syndrome and the ones present in our patient.

**Table 2:** Common abnormalities in WSS and their status in our patient ECG: electrocardiogram, MRI: magnetic resonance imaging, NT: not tested

Classical features	Status in our patient
Early-onset diabetes mellitus	Present
Primary amenorrhea	Present
Hypergonadotropic hypogonadism	Present
Intellectual disability	Present
Rudimentary fallopian tubes, hypoplastic uterus, streak ovaries	Present
Alopecia	Present
Facial features (prominent nasal base, prominent forehead, hypertelorism, progeroid appearance)	Present
Prominent ears	Absent
Movement disorder (dystonia, dysarthria, choreoathetosis, extrapyramidal symptoms)	Absent
Dental abnormalities (anodontia)	Absent
Sensorineural hearing loss	Not detected on bedside clinical assessment; audiometry not done
T-wave abnormalities in ECG	Absent
Brain MRI abnormalities	NT

The patient was initiated on estradiol replacement therapy for hypogonadism and associated osteopenia. Diabetes mellitus was managed with metformin, pioglitazone, and insulin, resulting in improved glycemic control. The patient and her family were provided with genetic counseling regarding the autosomal recessive inheritance of Woodhouse-Sakati syndrome, including the risk of recurrence in future offspring and the implications for family members. The importance of genetic testing for at-risk relatives and options for reproductive counseling, including carrier testing and prenatal diagnosis, were discussed.

## Discussion

The patient displays the classical triad of WSS: diffuse alopecia, hypogonadism, and diabetes mellitus, along with intellectual disability [2,4]. Notably, the presence of hypergonadotropic hypogonadism aligns with documented ovarian dysgenesis variants within WSS. Hepatic steatosis, although not traditionally emphasized, has been increasingly reported and may relate to metabolic dysfunction [7].

Management of Woodhouse-Sakati syndrome is primarily supportive and directed toward individual manifestations. Endocrine abnormalities are treated with standard therapies, including sex hormone replacement for hypogonadism to induce and maintain secondary sexual characteristics and preserve bone health, insulin or oral agents for diabetes mellitus, and levothyroxine for hypothyroidism. Recombinant IGF-1 therapy is not recommended due to a lack of demonstrated benefit. Neurological manifestations such as dystonia are managed with oral agents including anticholinergics, baclofen, and benzodiazepines, with botulinum toxin injections or, in refractory cases, deep-brain stimulation considered. Supportive care includes speech therapy for dysarthria, dietary modifications for dysphagia, audiological management for hearing loss, and educational support for intellectual disability. Regular surveillance is essential and includes periodic assessment of endocrine function (including glucose metabolism, thyroid function, and gonadal status), as well as neurological, developmental, and audiological evaluations [3].

Originally described in Middle East families with high rates of consanguinity in 1983, it has since been sporadically identified elsewhere, including in Europe, China, India, and Pakistan [4]. The first genetic identification of the pathologic gene mutation was by Alazami in 2008 with the identification of three different mutations affecting the *DCAF17* (formerly known as *C2orf37*) gene [1]. A total of at least 22 mutations have since been described in families with WSS (Table 3).

**Table 3:** DCAF17 mutations previously identified in families with WSS WSS: Woodhouse-Sakati syndrome

DCAF17 mutations identified in families with WSS	
1.	c.436delC (p.Ala147Hisfs*9) [4]
2.	c.1A>G (p.Met1?) [4]
3.	c.270delA (p.Lys90Asnfs*8) [4]
4.	c.321+1G>A [4]
5.	c.1091+1G>A [4]
6.	c.270dup (p.Cys91Metfs*28) [4]
7.	c.127-3delTAGinsAA [4]
8.	c.1423‐1_1425delGACA [4]
9.	c.1091+2T>C [4]
10.	c.1488_1489delAG [4]
11.	c.459-7_499del [4]
12.	c.1111delA [4]
13.	c.906G>A [4]
14.	c.1238delA [4]
15.	1422+5G [1]
16.	1091+6T>G [1]
17.	c.1001+1G>A [8]
18.	c.153G>A [9]
19.	c1422+3G>T [10]
20.	341C>A [11]
21.	387G>A [11]
22.	50delC (?) [1]

With the exception of c.436delC, a founder mutation identified in multiple families from the Arabian Peninsula and c.1091+1G>A, found in two separate families from Iran and Turkey, respectively, almost each of these is a private mutation found in a single consanguineous family.

WSS was first reported from India in 2008 by Koshy et al. [12], since which two more families have been reported [9,13]. Ours is the fourth such report from India, highlighting the higher relative incidence in this population. Consanguinity, which is a well-known risk factor for WSS, is much more prevalent in the Indian subcontinent than in Western populations, at least partly explaining the higher prevalence.

This specific mutation has previously been reported only once, in a family with WSS from the neighboring country of Pakistan [6]. The family had seven affected members with a similar phenotype. They did not report diabetes in any affected individual. This could be because all of them were young (ages 14-23), and the risk of diabetes in WSS is known to increase after early adulthood. In addition, they reported checking glycated hemoglobin (HbA1c) levels in only two patients. In all seven members, mild sensorineural hearing impairment was documented based on clinical assessment by the physician, without mention of formal audiometric evaluation; in contrast, no hearing loss was identified in our patient on bedside assessment. This difference may reflect phenotypic variability or the possibility of subtle hearing impairment that was not detected on history and bedside clinical assessment, as pure tone audiometry was not performed. Similar to our patient, most individuals in the series did not exhibit edentulism or extrapyramidal symptoms, although these features were present in a minority. All affected individuals, like our patient, had alopecia, moderate intellectual disability, hypogonadism, and characteristic dysmorphic facial features, including a high forehead, triangular face, prominent nasal root, and hypertelorism [6].

The mutation c.321+1G>A in the intron 3 of the *DCAF17* gene affects the canonical +1 donor splice site and is predicted to result in aberrant mRNA splicing and loss of normal protein function [4]. This variant has not been reported in population allele frequency databases including gnomAD (v4.1), gnomAD (v3.1), gnomAD (v2.1), 1000 genomes, and topmed. As discussed by Habib et al., this variant meets American College of Medical Genetics and Genomics (ACMG) criteria PVS1 (null variant in a gene where loss of function is a known mechanism) and PM2 (absent in population databases), supporting its classification as likely pathogenic [6]. It also fulfills the criteria PP4 (phenotype highly specific) and now PP5 (reputable source previously reported the variant as pathogenic), lending more support to its classification as likely pathogenic.

## Conclusions

This case highlights the classical clinical features of Woodhouse-Sakati syndrome and confirms the presence of the rare *DCAF17* c.321+1G>A splice-site variant through whole-exome sequencing. To our knowledge, this represents only the second reported occurrence of this mutation worldwide, providing independent evidence supporting its pathogenic role. Recognition of the characteristic phenotype, particularly the combination of alopecia, hypergonadotropic hypogonadism, diabetes mellitus, and intellectual disability, should prompt consideration of Woodhouse-Sakati syndrome and early genetic testing. The relatively higher prevalence of consanguineous marriages in regions such as Kashmir and the broader South Asian population may increase the likelihood of autosomal recessive disorders such as WSS, highlighting the importance of clinical awareness and access to genetic diagnostics in these settings. Additional reports of rare variants will be important for refining genotype-phenotype correlations and expanding the mutational spectrum of the *DCAF17* gene.
